# Aberrant Whole Blood Gene Expression in the Lumen of Human Intracranial Aneurysms

**DOI:** 10.3390/diagnostics11081442

**Published:** 2021-08-10

**Authors:** Vincent M. Tutino, Yongjun Lu, Daizo Ishii, Kerry E. Poppenberg, Hamidreza Rajabzadeh-Oghaz, Adnan H. Siddiqui, David M. Hasan

**Affiliations:** 1 Canon Stroke and Vascular Research Center, University at Buffalo, Buffalo, NY 14260, USA; vincentt@buffalo.edu (V.M.T.); kerrypop@buffalo.edu (K.E.P.); hrajabza@buffalo.edu (H.R.-O.); asiddiqui@ubns.com (A.H.S.); 2 Department of Pathology and Anatomical Sciences, University at Buffalo, Buffalo, NY 14260, USA; 3 Department of Neurosurgery, University at Buffalo, Buffalo, NY 14260, USA; 4 Department of Cardiovascular Medicine, University of Iowa Hospitals and Clinics, Iowa City, IA 52242, USA; yongjun-lu@uiowa.edu; 5 Department of Neurosurgery, University of Iowa Hospitals and Clinics, 1616 JCP, 200 Hawkins Dr, Iowa City, IA 52242, USA; daizo-ishii@uiowa.edu

**Keywords:** cerebral aneurysm, biomarkers, gene expression, transcriptomics, whole blood

## Abstract

The rupture of an intracranial aneurysm (IA) causes devastating hemorrhagic strokes. Yet, most IAs remain asymptomatic and undetected until they rupture. In the search for circulating biomarkers of unruptured IAs, we previously performed transcriptome profiling on whole blood and identified an IA-associated panel of 18 genes. In this study, we seek to determine if these genes are also differentially expressed within the IA lumen, which could provide a mechanistic link between the disease and the observed circulating gene expression patterns. To this end, we collected blood from the lumen of 37 IAs and their proximal parent vessels in 31 patients. The expression levels of 18 genes in the lumen and proximal vessel were then measured by quantitative polymerase chain reaction. This analysis revealed that the expression of 6/18 genes (*CBWD6*, *MT2A*, *MZT2B*, *PIM3*, *SLC37A3*, and *TNFRSF4*) was significantly higher in intraluminal blood, while the expression of 3/18 genes (*ST6GALNAC1*, *TCN2*, and *UFSP1*) was significantly lower. There was a significant, positive correlation between intraluminal and proximal expression of *CXCL10*, *MT2A*, and *MZT2B*, suggesting local increases of these genes is reflected in the periphery. Expression of *ST6GALNAC1* and *TIFAB* was significantly positively correlated with IA size, while expression of *CCDC85B* was significantly positively correlated with IA enhancement on post-contrast MRI, a metric of IA instability and risk. In conclusion, intraluminal expression differences in half of the IA-associated genes observed in this study provide evidence for IA tissue-mediated transcriptional changes in whole blood. Additionally, some genes may be informative in assessing IA risk, as their intraluminal expression was correlated to IA size and aneurysmal wall enhancement.

## 1. Introduction

Intracranial aneurysms (IAs) are present in 3–5% of the general population. They are a persistent societal and healthcare concern because, when they rupture, they cause devastating hemorrhagic strokes [1,2]. Aneurysmal subarachnoid hemorrhage carries high mortality (up to 50%) and morbidity (up to 50% among survivors) rates and high healthcare costs [3,4,5,6,7]. Early IA detection can enable periodic monitoring and preventive treatment aimed at reducing future ruptures [8]. However, as screening by medical imaging is prohibitively expensive and unduly risky, most unruptured IAs are often only incidentally detected on imaging for other medical reasons [9]. An inexpensive, blood-based diagnostic to identify individuals with unruptured IAs would facilitate a paradigm shift to proactive IA management via routine monitoring and preventive care.

In search of biomarkers in the blood, we hypothesized that molecular changes in circulating cells are associated with the presence of IA in the cerebral vasculature. In a series of studies [10,11,12,13], we performed transcriptome profiling on circulating blood constituents and found distinct transcriptional signatures of the disease, which have broadly been shown to reflect inflammatory cell activation, chemotaxis, and dysregulated inflammatory responses. Most recently, we performed transcriptome profiling of whole blood RNA from n = 34 patients with IA and n = 33 IA-free controls (confirmed on angiography) and identified a panel of 18 genes that distinguished IA patients with an accuracy of 85% and area under the receiver operating characteristic curve of 0.91 in an independent validation cohort [14]. These findings led us to ask the question, “what is the source of dysregulated gene expression in the blood of patients with IA?”.

To answer this question, we sought to determine if an interaction between blood cells and the diseased aneurysmal tissue, via contact or through factors released into the blood, could propagate gene expression changes in circulating cells [15]. To this end, we analyzed expression of the 18 genes in blood collected intraluminally, from inside the IA sac, and from circulating blood in the parent artery, proximal to the aneurysm. We also explored if luminal or peripheral expression of the 18 genes was related to IA size (the preeminent metric to assess aneurysm rupture risk [16]) and instability, as assessed by vessel wall enhancement (VWE) on post-contrast magnetic resonance imaging (MRI). We hope these findings can begin to shed light on the basis of dysregulated gene expression that has been observed in the blood of patients with IA.

## 2. Methods

### 2.1. Study Participants

The study protocol was approved by the University of Iowa Institutional Review Board (study number 201905780). Written informed consent was obtained from all subjects prior to sample collection, and the study was carried out in accordance with the approved protocol. Consecutive adult patients presenting to the Department of Neurosurgery at the University of Iowa Hospitals and Clinics between October 2019 and January 2021 who underwent IA treatment via coiling or WEB device, and who received contrast-enhanced MRI (as described elsewhere [17]), were prospectively enrolled in this study. We excluded patients with previously-treated IAs and those taking corticosteroids or immunosuppressant medications. Information about patient’s history and comorbidities was also collected from electronic medical records.

### 2.2. Intra-Aneurysmal Blood Collection

The technique for intraluminal blood sampling has been previously described [15,18,19]. In brief, arterial blood samples were collected endovascularly via catheter during digital subtraction angiography. One 10 mL blood sample was collected via guide catheter from the ipsilateral parent artery proximal to the IA. Then, a 3 mL blood sample was collected intraluminally, via a microcatheter positioned inside the IA sac prior to coil treatment. All samples were collected into BD Vacutainer Glass Whole Blood Tubes (BD, Franklin Lakes, NJ, USA) containing a 1.5 mL anticoagulant solution of trisodium citrate (22.0 g/L), citric acid (8.0 g/L), and dextrose (24.5 g/L).

### 2.3. Sample Processing and RNA Extraction

After collection, blood samples were centrifuged to remove the plasma, and cellular components were frozen at −80 °C until processing. Before RNA isolation, cells were thawed, resuspended in Hank’s balanced salt solution (Thermo Fisher Scientific, Waltham, MA, USA), and centrifuged. The interface layer containing leukocytes and erythrocytes was isolated and erythrocytes were subsequently removed by washing with lysis buffer. The leukocyte pellet was then disrupted in 0.2 mL of Trizol solution (Life Technologies, Carlsbad, CA, USA) and disrupted by a pellet pestle (RPI), after which an additional 0.8 mL was added. Total RNA was then extracted from Trizol, according to the manufacturer’s instructions, and trace DNA was removed via Ambion’s DNase Treatment Kit (Thermo Fisher Scientific, Waltham, MA, USA). The purity and concentration of each sample were assessed by measuring absorbance at 230 nm, 260 nm, and 280 nm on a NanoDrop 1000 (Thermo Fisher Scientific, Waltham, MA, USA).

### 2.4. Quantitative Polymerase Chain Reaction (qPCR) Analysis

The expression of the 18 previously-identified genes (*ATF3*, *CBWD6*, *CCDC85B*, *CCR8*, *CHMP4B*, *CLEC4F*, *CXCL10*, *FN1*, *MT2A*, *MZT2B*, *PCSK1N*, *PIM3*, *SLC37A3*, *ST6GALNAC1*, *TCN2*, *TIFAB*, *TNFRSF4*, and *UFSP1*) was assessed in all samples by qPCR. *GAPDH* was used as the housekeeping gene and assayed in parallel. Gene-specific, oligonucleotide primer sequences were based on data from OriGene Technologies (https://www.origene.com/, accessed November 2020), with the exception of those for *GAPDH*, which was designed in-house via Primer Designer v4.20 (Sci Ed Central, Cary, NC, USA). All primers (IDT) had a melting temperature of ~60–64 °C, a length of 15–25 nucleotides, and produced PCR products with lengths of 50–200 base pairs. The target specificity for each primer pair was verified via Primer BLAST (National Center for Biotechnology Information) and the replication efficiency of each pair was assessed as previously described. All primers pairs were specific to their target and had sufficient efficiency (0.9–1.1) [20]. Primer sequences, annealing temperatures, and product lengths are shown in Supplemental Table S1. For reverse transcription, we used the AffinityScript QPCR cDNA Synthesis kit (Agilent Technologies, Santa Clara, CA, USA) according to the manufacturer’s instructions.

Quantitative PCR was run with 10 ng of cDNA and 0.5 μM of each primer pair in 25 μL reactions on the Bio-Rad CFX machine (Bio-Rad, Hercules, CA, USA) using the Brilliant II SYBR Green qPCR Master Mix (Agilent Technologies, Santa Clara, CA, USA), according to the manufacturer’s instructions. The temperature profile consisted of an initial step of 95 °C for 10 min, followed by 40 cycles of 95 °C for 30 s and 60 °C for 1 min. This was followed by one cycle step for a dissociation curve, with 95 °C for 15 s, plus a dissociation cycle with 95 °C for 15 s, 60 °C for 1 min, and 95 °C for 15 s. Gene-specific amplification was confirmed by a single peak using the Bio-Rad dissociation melt curve. Undetectable expression at cycle > 40 was given a C_t_ value of 41 for subsequent analysis. *GAPDH* expression was used for normalization, and the relative expression levels were calculated for each gene using the 2^−ΔΔCt^ method.

### 2.5. Correlation with IA Size and Instability

We explored the relationship between expression and IA size, and expression and aneurysmal VWE. Pearson correlation analysis was performed between intraluminal and parent vessel gene expression; maximum IA size was quantified on 2D digital subtraction angiography. We also performed Pearson correlation analysis between transcripts per million (TPM) normalized whole blood sequencing data of the 18 genes (from our previous study, GSE159610, with n = 34 patients with unruptured IAs) [14]. For correlation with VWE, enhancement was quantified by calculating CR_stalk_, a previously-described parameter that is the maximal post-contrast MRI intensity in the IA wall, normalized to the intensity of the pituitary stalk [17,21]. CR_stalk_ has been previously demonstrated to be an objective and validated metric of IA instability [17]. To quantify the degree of association between gene expression and CR_stalk_, we again performed Pearson correlation analysis.

### 2.6. Statistical Analysis

To assess expression differences between two conditions (i.e., between blood in the IA sac and in the proximal parent artery), we first evaluated distribution normality using the Shapiro–Wilk test. Normally distributed expression levels were compared using a Student’s *t*-test. Non-normally distributed data were compared using a Mann–Whitney U-test. Any difference was considered statistically significant if *p* < 0.05. For correlation analyses, Pearson correlation was assessed as previously described [22]. To measure the degree of correlation, we assessed the Pearson correlation coefficient (PCC) and *p*-value from the Wald test. An absolute 1 ≥ PCC ≥ 0.80 represented “very strong” correlation, 0.79 ≥ |PCC| ≥ 0.60 represented “strong” correlation, 0.59 ≥ |PCC| ≥ 0.40 represented “moderate” correlation, 0.39 ≥ |PCC| ≥ 0.20 represented “weak” correlation, and |PCC| < 0.19 represented “very weak” or no correlation [23].

## 3. Results

### 3.1. Study Participants

We analyzed blood samples from 31 patients who had 37 IAs (4 had multiple IAs). As shown in Table 1, the average age of the study participants was 63.0 years. In all, 80.6% were female, 61.3% were current smokers, 77.4% had hypertension, 45.2% had hyperlipidemia, and 6.5% were diabetic. A total of 37 intraluminal blood RNA samples were compared against 33 unique proximal parent vessel blood RNA samples, as three cases had multiple, ipsilateral IAs that shared a parent artery. Table 2 shows additional information about each IA. Isolated RNA was of high quality, with an average 260/280 = 1.9, an average 260/230 = 1.62, and an average concentration = 384.7 mg/µL. See Supplemental Table S2 for RNA quality and quantity data.

### 3.2. Differential Expression in the IA Sac versus the Proximal Parent Vessel

**Figure 1:**
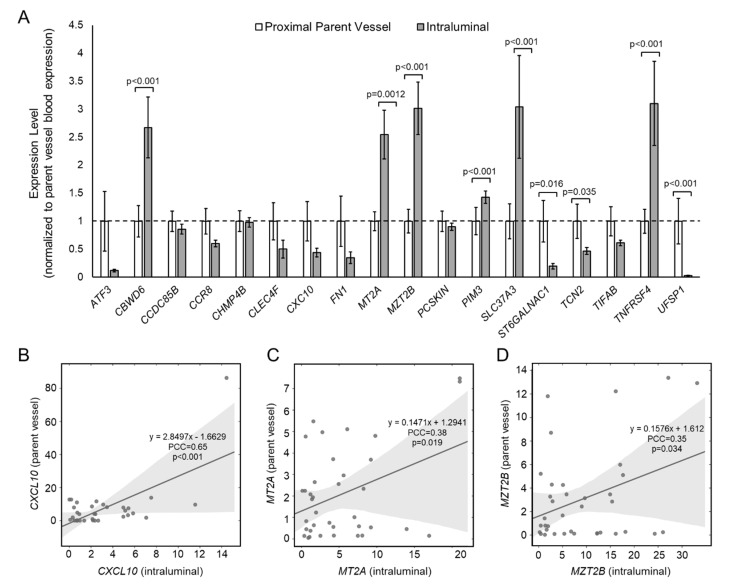
Expression of 18 IA-associated genes in IA sac and proximal parent vessel. (**A**) A bar graph showing 2^−ΔΔCt^ expression levels of whole blood RNAs in the proximal parent vessel and the IA sac. All data are normalized to parent vessel expression levels and error bars represent standard error. In all, six genes had significantly higher expression in IA sac, while three genes had significantly lower expression in IA sac. (**B**–**D**) Pearson correlation analysis showed that intraluminal expression was significantly positively correlated with expression in the parent vessel for *CXCL10* (**B**), *MT2A* (**C**), and *MZT2B* (**D**), as all had PCC > 0.3 (abbreviations: IA = intracranial aneurysm, PCC = Pearson correlation coefficient).

To determine if aberrant gene expression was due, at least in part, to an interaction between blood cells and the IA tissue, we tested expression of the 18 panel genes in intraluminal IA blood and in blood from the proximal parent vessel. Figure 1A shows differential gene expression results from qPCR analysis. The mean expression levels of *CBWD6*, *MT2A*, *MZT2B*, *PIM3*, *SLC37A3*, and *TNFRSF4* were statistically significantly higher in intraluminal blood, and the mean expression levels of *ST6GALNAC1*, *TCN2*, and *UFSP1* were statistically significantly lower in the intraluminal blood. All other genes, most of which had lower expression in the IA sac, were not significantly differentially expressed between blood in the IA sac and in the parent vessel (see Supplemental Table S3 for fold-change and *p*-values of differential expression). Figure 1B–D demonstrates significant Pearson correlations between intraluminal expression and expression in the parent vessel. There was a significant, positive correlation between intraluminal and parent vessel expression of *CXCL10* (Pearson correlation coefficient (PCC) = 0.65, a strong correlation), *MT2A* (PCC = 0.38, a weak correlation), and *MZT2B* (PCC = 0.35, a weak correlation), suggesting local increase of these genes was reflected by an increase in the peripheral blood. See Supplemental Table S4 for PCCs and *p*-values for this correlation analysis.

### 3.3. Correlation between IA Size and Intraluminal Gene Expression

**Figure 2:**
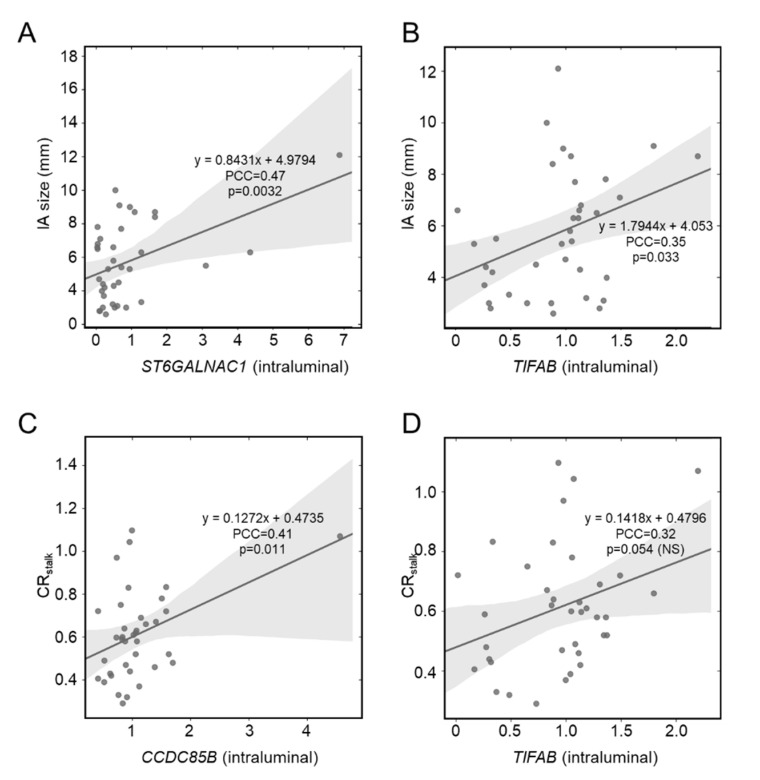
Pearson correlation analysis of whole blood gene expression and IA risk metrics. (**A**,**B**) Intraluminal expression of *ST6GALNAC1* (**A**) and *TIFAB* (**B**) was significantly positively correlated with IA size. There was no correlation between proximal parent vessel blood gene expression and IA size. (**C**,**D**) Intraluminal expression of *CCDC85B* (**C**) was significantly positively correlated with aneurysmal VWE, as quantified by CR_stalk_ (PCC > 0.3). There was also a positive correlation between *TIFAB* (**D**) and CR_stalk_ (PCC > 0.3), albeit the correlation was not significant. There was no correlation between proximal parent vessel blood gene expression and aneurysmal wall enhancement (abbreviations: IA = intracranial aneurysm, NS = not significant, PCC = Pearson correlation coefficient, VWE = vessel wall enhancement).

Previously, we showed expression differences in circulating inflammatory cells, namely neutrophils, were exaggerated in patients with larger IAs [10,11]. Pearson correlation analysis demonstrated that intraluminal expression of *ST6GALNAC1* (PCC = 0.47, a moderate correlation) and *TIFAB* (PCC = 0.35, a weak correlation) had a significant positive correlation with IA size (Figure 2A,B). However, there were no significant correlations between expression levels in the proximal parent artery and IA size. We found no overlap in significant correlates in the gene expression data from our previous study. In our RNA sequencing data, there were significant positive correlations between IA size and *CHMP4B* (PCC = 0.45, a moderate correlation), *MZT2B* (PCC = 0.38, a weak correlation), *PCSK1N* (PCC = 0.35, a weak correlation), and *PIM3* (PCC = 0.35, a weak correlation). Interestingly, the correlation trends (positive or negative) in our sequencing data were more similar to that of the proximal parent vessel. This may be because both data were from peripheral arterial blood. See Supplemental Table S5 for PCCs and *p*-values for all correlation analyses with IA size.

### 3.4. Correlation between Aneurysmal VWE and Intraluminal Gene Expression

Increased VWE of the IA has recently emerged as a metric for aneurysm instability and risk [17] and has been found to be related to degeneration of the vascular tissue and inflammation [24,25,26]. Therefore, we suspected that there may greater interaction of circulating blood cells with the damaged aneurysm tissue or released cytokines/chemokines in the lumen of enhancing IAs. As shown in Figure 2C, Pearson correlation analysis demonstrated that intraluminal expression of only *CCDC85B* (PCC = 0.41, a moderate correlation) had a significant positive correlation with IA VWE, as quantified by CR_stalk_ (a metric of maximal, normalized post-contrast MRI intensity), although *TIFAB* did have a PCC > 0.3 (Figure 2D). There was no correlation between proximal parent vessel blood expression and aneurysmal VWE. See Supplemental Table S6 for PCCs and *p*-values for all correlation analyses with CR_stalk_.

## 4. Discussion

In previous work, we identified a panel of 18 genes in circulating whole blood that were significantly differentially expressed in patients with IAs compared with IA-free controls [14]. Bioinformatics analyses indicated that critical inflammatory behaviors, namely regulation by NF-κB (a key transcription factor in IA natural history [27,28]), were represented by this combination of genes. However, the source of these aberrant gene expression changes was unclear. Differential gene expression could be induced by contact with the diseased IA tissue. The aneurysmal tissue (that can be lined with intraluminal thrombi or atherosclerotic plaques) is characterized by escalating inflammatory responses and progressive vascular degeneration [29,30,31,32], which is aided by elevated levels of proteinases and reactive oxygen species [33,34,35]. Furthermore, the IA wall may locally release chemokines and chemoattractant cytokines (such as IL-1, IL-8, and IL-17), which can peripherally activate circulating immune cells in the blood [15]. Alternatively, aberrant gene expression may be inherent, resulting from heritable genetic factors (something we have explored in separate studies [36,37]), or could even be promulgated by combinations of risk factors that are commonly associated with IA.

In this study, we sought to test if the expression of these 18 genes was related to cellular contact with the IA wall, or with other factors released from the diseased aneurysmal tissue. This would provide a mechanistic link between the aneurysm disease and observed circulating gene expression patterns. Therefore, we independently assessed if our previously-identified gene panel was more highly or lowly expressed within the aneurysm lumen. Our data demonstrated that half (9/18) of the IA-associated genes were significantly differentially expressed in the blood from the IA lumen. We observed statistically significant increases in *CBWD6*, *MT2A*, *MZT2B*, *PIM3*, *SLC37A3*, and *TNFRSF4* expression and statistically significant decreases in *ST6GALNAC1*, *TCN2*, and *UFSP1* expression in the IA lumen. These findings provide the first evidence of localized gene expression changes in circulating blood cells within the sac of the aneurysm lesion.

In our previous study, these genes (with the exception of *MT2A* and *TCN2*) were found to be upregulated in systemic whole blood in patients with IA compared with IA-free controls. The current findings may thus indicate that local increases in *CBWD6*, *MZT2B*, *PIM3*, *SLC37A3*, and *TNFRSF4* could also be detectable in the peripheral blood. Studies show that, after aneurysm genesis, the enlargement of the IA sac exposes the wall to increasingly static flow and lower wall shear stress. We suspect that sluggish aneurysm flow provides sufficient time for leukocyte interaction with the diseased IA wall and with chemokines and chemoattractant cytokines secreted by mural cells. Chalouhi et al. [15] found that plasma levels of MCP-1 [38], RANTES [39], MIG [40], Eotaxin [41], IL-8 [42], and IL-17 [43] were increased in unruptured intracranial aneurysms. These proteins are all activated by, or stimulate, the NF-kB pathway, which has been shown to regulate inflammatory signaling in aneurysm pathogenesis [28,44].

We suspect that coordination of, and regulation by, canonical and non-canonical NF-kB pathways may also be related to local increased expression of *TNFRSF4*, *MT2A*, and *PIM3* that we observed in our analysis [45]. Indeed, *TNFRSF4*, or *OX40*, encodes a ligand receptor of the TNF superfamily that is typically expressed by T-cells 24–72 h after activation [46,47,48,49,50,51,52,53]. This receptor has been shown to activate NF-kB via its interaction with adaptor proteins TRAF2 and TRAF5 (non-canonical). On the other hand, *MT2A* helps regulate NF-kB activity by modulating expression of IkB-a and has been shown to play a role in transmitting inflammatory signals to the vascular endothelium [54,55]. *PIM3*, which encodes a serine/threonine kinase, is one of several regulatory kinases that can be induced by NF-kB, which functions upstream of IkB-a and can lead to further activation of NF-kB itself (canonical) [56,57]. These findings suggest a complex cellular environment within the lumen of unruptured IAs, in which inflammatory pathways, potentially activated by MCP-1, RANTES, MIG, Eotaxin, IL-8, and IL-17, are regulated through NF-kB mediated transcription and downstream signal transduction.

Our correlation analyses also implicated local inflammatory signaling through NF-kB in leukocyte expression in larger and enhancing IAs. For example, *ST6GALNAC1* expression in the IA sac was significantly positively correlated with IA size. *ST6GALNAC1* encodes a sialyltransferase involved in protein glycosylation and is activated by CCL17 through the NF-kB pathway signaling [58,59,60]. *TIFAB*, which encodes a regulator of TRAF proteins that transduce extracellular inflammatory signals that mediate the NF-kB pathway, was also positively correlated with aneurysm size (and with IA wall enhancement, albeit not significantly) [61,62]. The only gene correlated with CR_stalk_ was *CCDC85B* (intraluminal), which encodes coiled-coil domain protein that functions in several physiological processes, such as regulation of signal transduction, gene expression, cell division, and cell motility [63]. Based on this analysis, we suspect that expression of *ST6GALNAC1*, *TIFAB*, and *CCDC85B* may also be a good candidate marker for identifying higher risk IAs, as both size and aneurysm wall enhancement are associated with IA instability and rupture. Interestingly, there was no correlation between IA size or VWE and proximal parent vessel blood expression, suggesting that such a relationship may only be detectable in close proximity to the aneurysmal lesion, at least for the genes we analyzed here.

This study has several limitations. First, it is a single-center study, which may have introduced selection bias in our experimental design. However, it is intended to be an external validation of the supposed source of gene expression differences identified in our previous study, which was performed at another center. Second, it is unclear if the presence of comorbidities and other confounding factors contributed to differential expression. We believe that this is unlikely, as the study was internally controlled; whole blood expression within each IA was compared to that within the IA’s respective proximal parent vessel. Third, most of the significant correlations observed in this study were either weak or moderate (with PCC < 0.60). Future studies in larger datasets could provide greater statistical power in assessing correlation strength. Lastly, several of the differentially expressed genes in this study were related to NF-kB, leading us to hypothesize a pivotal role of this transcription factor in peripheral activating of circulating immune cells in IA. However, experimental studies will be needed to test this hypothesis.

## 5. Conclusions

In this study, we demonstrated that nine genes of a previously-identified IA-associated circulating blood gene panel are differentially expressed in the lumen of aneurysm lesions. For the first time, this provides evidence for aneurysm tissue-mediated transcriptional changes in peripheral blood cells. Based on our analysis, these changes appear to be related to various facets of canonical and non-canonical NF-kB signaling. Furthermore, our Pearson correlation analysis showed that some of genes (namely, *ST6GALNAC1*, *TIFAB*, and *CCDC85B*) may be informative in assessing IA risk, as their intraluminal expression was related to aneurysm size, IA wall enhancement, or both.

## 6. Patents

The 18 biomarker genes were included as part of a provisional patent filed in September 2020.

## Figures and Tables

**Table 1:** Patient characteristics *.

Characteristic	Value
Age (average years ± SD)	63.0 ± 11.7
Female gender (n/n_total_)	25/31 (80.6%)
Smoking (n/n_total_)	19/31 (61.3%)
Hypertension (n/n_total_)	24/31 (77.4%)
Hyperlipidemia (n/n_total_)	14/31 (45.2%)
Diabetes mellitus (n/n_total_)	2/31 (6.5%)
Patients with multiple IAs (n/n_total_)	4/31 (12.9%)
Total number of IAs (n/n_total_)	37/31
IA location (n/n_total_IA_)	
ACA	1/37 (2.7%)
ACom	7/37 (18.9%)
BT	5/37 (13.5%)
ICA	14/37 (37.8%)
MCA	6/37 (16.2%)
PCom	2/37 (5.4%)
PICA	1/37 (2.7%)
VA	1/37 (2.7%)

* Abbreviations: ACA = anterior cerebral artery, ACom = anterior communicating artery, BT = basilar terminus, IA = intracranial aneurysm, ICA = internal carotid artery, MCA = middle cerebral artery, n = number, PCom = posterior communicating artery, PICA = posterior inferior cerebellar artery, SD = standard deviation, VA = vertebral artery.

**Table 2:** Aneurysm characteristics *.

Pt. ID	IA ID	PV ID	Location	Max. D	CR_stalk_
1	IA1	PV1	R MCA	7.8	0.58
2	IA2	PV2	R ICA (term.)	8.7	1.07
3	IA3	PV3	R ACA	4.0	0.52
4	IA4	PV4	BT	7.1	0.72
5	IA5	PV5/6	R ICA (paraop.)	6.5	0.58
5	IA6	PV5/6	R ICA (paraop.)	6.3	0.46
5	IA7	PV7	L ICA (Op.)	2.8	0.69
6	IA8	PV8	R ICA (term.)	3.1	0.52
7	IA9	PV9	ACom	5.8	0.39
8	IA10	PV10	L PCom	8.4	0.83
9	IA11	PV11	L ICA (term.)	6.6	0.63
10	IA12	PV12	ACom	5.3	0.47
11	IA13	PV13	ACom	9.0	0.97
12	IA14	PV14	BT	3.3	0.32
13	IA15	PV15	ACom	3.2	0.61
14	IA16	PV16	R ICA	5.4	0.78
15	IA17	PV17	L ICA (op.)	3.7	0.59
16	IA18	PV18	L ICA (op.)	4.7	0.37
17	IA19	PV19/20	L ICA	9.1	0.66
17	IA20	PV19/20	L MCA	4.4	0.48
18	IA21	PV21	PICA	4.3	0.42
19	IA22	PV22	R MCA	5.5	0.33
20	IA23	PV23	R PCom	7.7	0.49
21	IA24	PV24	R ICA (paraop.)	3.0	0.62
22	IA25	PV25	ACom	2.8	0.43
23	IA26	PV26	ACom	8.7	0.60
24	IA27	PV27/28/29	R MCA	2.6	0.64
24	IA28	PV27/28/29	R MCA	3.0	0.44
24	IA29	PV27/28/29	R ICA	3.0	0.75
25	IA30	PV30	BT	12.1	1.10
26	IA31	PV31	BT	5.3	0.41
27	IA32	PV32	R MCA	6.6	0.72
28	IA33	PV33	ACom	4.5	0.29
29	IA34	PV34	BT	10.0	0.67
30	IA35	PV35	R ICA (paraop.)	4.2	0.83
31	IA36	PV36	R ICA (paraop.)	6.8	0.60
31	IA37	PV37	L. VA	6.3	1.04

* Abbreviations: ACA = anterior cerebral artery, ACom = anterior communicating artery, BT = basilar terminus, D = diameter, IA = intracranial aneurysm, ICA = internal carotid artery, ID = identification number, L = left, Max. = maximum, MCA = middle cerebral artery, n = number, op. = ophthalmic, paraop. = paraopthalmic, PCom = posterior communicating artery, PICA = posterior inferior cerebellar artery, Pt. = patient, PV = parent vessel, R = right, SD = standard deviation, term. = terminus, VA = vertebral artery.

## Data Availability

All data relevant to the qPCR experiments are present in the manuscript and the Supplementary Material. Next-generation RNA sequencing data from our previous whole blood expression profiling study can be found at NCBI’s GEO (accession No. GSE159610).

## Supplementary Materials

The following are available online at https://www.mdpi.com/article/10.3390/diagnostics11081442/s1: Supplemental Table S1: Primers Used for qPCR; Supplemental Table S2: RNA Quality and Quantity; Supplemental Table S3: Differential Expression Between Genes in the IA Sac and Genes in the Proximal Parent Vessel; Supplemental Table S4: Correlation Between Expression in the IA Sac and Expression in the Proximal Parent Vessel; Supplemental Table S5: Correlation Between Gene Expression and IA Size; Supplemental Table S6: Correlation Between Gene Expression and IA CR_stalk_.
