# Supplementary material for: Aberrant Whole Blood Gene Expression in the Lumen of Human Intracranial Aneurysms
Source: Diagnostics (Basel). 2021 Aug 10;11(8):1442. doi: 10.3390/diagnostics11081442 (PMC8392298; doi:10.3390/diagnostics11081442)
Supplement: Supplementary file 1 [file diagnostics-11-01442-s001.zip › diagnostics-1311525-supplementary.pdf]

Supplemental Information for: Aberrant Whole Blood Gene Expression in the Lumen of Human Intracranial Aneurysms

Vincent M. Tutino PhD[1,2,3], Yongjun Lu PhD[4], Daizo Ishii MD[5], Kerry E. Poppenberg PhD[1,3], Hamidreza Rajabzadeh-Oghaz PhD[1,3], Adnan H. Siddiqui MD-PhD[1,3], *David M. Hasan MD[5]

[1]Canon Stroke and Vascular Research Center; [2]Department of Pathology and Anatomical Sciences, [3]Department of Neurosurgery, University at Buffalo, Buffalo, NY, USA
[4]Departmet of Cardiovascular Medicine, [5]Department of Neurosurgery, University of Iowa Hospitals and Clinics, Iowa City, IA, USA

***Corresponding Author:**
David Hasan, MD
Department of Neurosurgery
1616 JCP
200 Hawkins Dr
Iowa City, IA 52242
E-mail: david-hasan@uiowa.edu
Phone: 319-384-8669

Supplemental Tables

Supplemental Table S1. Primers Used for qPCR.*

Gene/ GenBank Acc. #	Primer Sequence	Avg. Annealing Temp. (°C)	PCR Prod. Length (bp)
ATF3	5'-CGCTGGAATCAGTCACTGTCAG-3'	62.5	137
NM_001674	5'-CTTGTTTCGGCACTTTGCAGCTG-3'		
CBWD6	5'-GAAACGGTTGCCTCTGCTGTTC-3'	62.0	119
NM_001085457.2	5'-AGGGTCTGCTAATCCAGTGGTC-3'		
CCDC85B	5'-TCATGCAGGAGGTGAATCGGCA-3'	64.1	123
NM_006848	5'- AGTCCAGGAAGCAGCAGAGGTC-3'		
CCR8	5'-TGGCTGTTGTCCATGCCGTGTA-3'	63.6	104
NM_005201	5'- TGGGATGGTAGCCATAATGGCG-3'		
CHMP4B	5'- ACCAACACCGAGGTGCTCAAGA -3'	63.3	127
NM_176812	5'-CTGCAAGTTCTTGCTGGTCAGC-3'		
CLEC4F	5'-CCAAGATACCGAGGCTCGTTCA-3'	62.3	116
NM_173535	5'-AGGCTTCGGAACAGGTCTTGTC-3'		
CXCL10	5'-GGTGAGAAGAGATGTCTGAATCC-3'	60.7	134
NM_001565	5'-GTCCATCCTTGGAAGCACTGCA-3'		
FN1	5'-ACAACACCGAGGTGACTGAGAC-3'	61.8	143
NM_212482	5'-GGACACAACGATGCTTCCTGAG-3'		
MT2A	5'-GAGTGCAAATGCACCTCCTGCAA-3'	63.1	156
NM_005953	5'-GCGTTCTTTACATCTGGGAGCG-3'		
MZT2B	5'-AGGTGCTGAGCACCGAGGAGA-3'	64.9	102
NM_025029	5'-GCTTCAGCAGGTCCACCAGGA-3'		
PCSK1N	5'-AGGAGGCAGGCGACGAGACAC-3'	65.6	102
NM_013271.5	5'-GCTCAGAGCCCACATCGTGGT-3'		
PIM3	5'-GGACAAGGAGAGCTTCGAGAAG-3'	61.7	147
NM_001001852	5'-CTCCTTCACCACGTGCTTCACA-3'		
SLC37A3	5'-GTGGGCAACATTTTGGGAGCGT-3'	63.7	129
NM_032295	5'-TCTGGTGACACCAGGAGTCCAA-3'		
ST6GALNAC1	5'-CTCTCTTCCTGGACTCCAGACA-3'	62.7	134
NM_018414	5'-AAGCGTGTCACGACCTTCTGCA-3'		
TCN2	5'-CAGAACAGTGCGAGAGGAGATC-3'	61.8	121
NM_000355	5'-TCGCCTTGAGACATGCTGTTCC-3'		
TIFAB	5'-CTCCTTCTCAGGCATCCAGATG-3'	60.9	123
NM_001099221	5'-CCATTCGTCAGTTTCCTCAGCC-3'		
TNFRSF4	5'-ACAACGACGTGGTCAGCTCCAA-3'	64.8	112
NM_003327	5'-CAGCGGCAGACTGTGTCCTGT-3'		

UFSP1<br>NM_001015072	5'-GGAGAGGCTTTACTCGCACTTC-3'<br>5'-CCAATACCAGGACATAGGCTTCC-3'	60.7	131
GAPDH (HK)<br>NM_002046	5'-AGCTCACTGGCATGGCCTTC-3'<br>5'-CGCCTGCTTCACCACCTTCT-3'	62.8	116

*All primer pairs had estimated efficiency ranging from 0.9-1.1. (Acc.=accession, Avg.=average, bp=base pair, HK=housekeeping, Prod.=product, qPCR=quantitative polymerase chain reaction, Temp.=temperature)

Supplemental Table S2. RNA Quality and Qunatity.*

Sample ID	260/280	260/230	Conc. (ng/uL)
IA1	1.95	1.65	155
IA2	2.03	1.6	401
IA3	2.04	1.66	420
IA4	1.97	1.68	426
IA5	1.91	1.58	613
IA6	1.92	1.73	499
IA7	1.95	1.64	512
IA8	1.99	1.66	450
IA9	1.83	1.64	380
IA10	1.97	1.68	355
IA11	1.83	1.63	592
IA12	1.82	1.57	325
IA13	1.84	1.57	191
IA14	1.89	1.62	620
IA15	1.88	1.61	501
IA16	1.88	1.63	510
IA17	1.87	1.64	712
IA18	1.83	1.57	505
IA19	1.83	1.57	429
IA20	1.82	1.64	598
IA21	1.86	1.63	230
IA22	1.81	1.59	196
IA23	1.85	1.58	240
IA24	1.89	1.58	131
IA25	2.03	1.71	360
IA26	1.92	1.64	334
IA27	1.95	1.74	367
IA28	1.87	1.62	724
IA29	1.82	1.71	180
IA30	1.89	1.61	627
IA31	1.89	1.62	189
IA32	1.82	1.59	126
IA33	1.92	1.66	340
IA34	1.87	1.6	510
IA35	1.85	1.55	588
IA36	1.87	1.63	556
IA37	1.86	1.62	256
PV1	1.95	1.65	305
PV2	1.98	1.61	193

PV3	1.97	1.57	590
PV4	2.00	1.61	352
PV5/6	1.97	1.67	466
PV7	2.06	1.62	398
PV8	1.86	1.55	592
PV9	1.90	1.57	140
PV10	1.83	1.55	144
PV11	2.01	1.67	261
PV12	1.85	1.57	130
PV13	2.10	1.68	235
PV14	2.04	1.61	544
PV15	1.89	1.55	617
PV16	1.85	1.57	136
PV17	1.87	1.56	136
PV18	1.88	1.58	196
PV19/20	1.95	1.62	401
PV21	1.86	1.57	668
PV22	1.81	1.55	662
PV23	1.85	1.49	461
PV24	1.80	1.59	521
PV25	2.10	1.63	414
PV26	1.85	1.56	655
PV27/28/29	1.98	1.59	578
PV30	1.84	1.64	550
PV31	1.92	1.58	219
PV32	1.90	1.64	121
PV33	1.83	1.66	213
PV34	1.82	1.62	191
PV35	1.83	1.64	151
PV36	1.85	1.66	172
PV37	1.97	1.62	368

*All RNA samples were of sufficient quality and quantity for our analyses. (Conc.=concentration, IA=intracranial aneurysm, ID=identification number, PV=parent vessel)

Supplemental Table S3: Differential Expression Between Genes in the IA Sac and Genes in the Proximal Parent Vessel.*

Gene	Log2(F-C)	p-value
ATF3	-3.091	0.099
CBWD6	1.419	<0.001†
CCDC85B	-0.225	0.496
CCR8	-0.731	0.090
CHMP4B	-0.031	0.113
CLEC4F	-0.996	0.163
CXCL10	-1.189	0.269
FN1	-1.528	0.385
MT2A	1.349	0.001†
MZT2B	1.592	<0.001†
PCSKIN	-0.154	0.182
PIM3	0.512	<0.001†
SLC37A3	1.605	<0.001†
ST6GALNAC1	-2.346	0.016†
TCN2	-1.097	0.035†
TIFAB	-0.709	0.087
TNFRSF4	1.634	<0.001†
UFSP1	-5.081	<0.001†

*Note: † denotes significant p-value (<0.05). (F-C=fold-change)

Supplemental Table S4: Correlation Between Expression in the IA Sac and Expression in the Proximal Parent Vessel.*

Gene	PCC	p-value
ATF3	-0.070	0.679
CBWD6	0.048	0.779
CCDC85B	-0.042	0.805
CCR8	-0.080	0.636
CHMP4B	0.090	0.598
CLEC4F	-0.019	0.910
CXCL10	0.649	<0.001†
FN1	0.018	0.918
MT2A	0.385	0.019†
MZT2B	0.349	0.034†
PCSK1N	-0.164	0.333
PIM3	-0.281	0.092
SLC37A3	0.285	0.087
ST6GALNAC1	0.105	0.536
TCN2	-0.142	0.403
TIFAB	-0.158	0.350
TNFRSF4	-0.071	0.678
UFSP1	-0.168	0.319

*Note: † denotes significant p-value (<0.05). (PCC=Pearson correlation coefficient)

Supplemental Table S5: Correlation Between Gene Expression and IA Size.*

	Intraluminal		Proximal Parent Vessel		RNA-Seq (GSE159670)	
**Gene**	**PCC**	**p-value**	**PCC**	**p-value**	**PCC**	**p-value**
ATF3	-0.216	0.198	0.110	0.515	0.095	0.590
CBWD6	-0.235	0.162	0.105	0.538	-0.007	0.970
CCDC85B	0.185	0.274	0.015	0.931	0.330[‡]	0.055
CCR8	0.261	0.119	-0.020	0.909	0.210	0.220
CHMP4B	-0.266	0.111	0.046	0.786	0.450	0.007[†]
CLEC4F	-0.003	0.984	0.024	0.887	0.056	0.750
CXCL10	-0.173	0.306	0.031	0.856	-0.110	0.550
FN1	-0.197	0.242	-0.103	0.544	-0.030	0.870
MT2A	-0.240	0.153	0.123	0.469	0.160	0.360
MZT2B	-0.141	0.406	0.103	0.544	0.380	0.027[†]
PCSK1N	-0.014	0.935	0.080	0.637	0.350	0.041[†]
PIM3	-0.039	0.817	0.136	0.422	0.350	0.044[†]
SLC37A3	-0.150	0.376	0.090	0.598	0.017	0.920
ST6GALNAC1	0.471	0.003[†]	-0.028	0.867	0.021	0.900
TCN2	0.153	0.368	0.050	0.769	-0.055	0.760
TIFAB	0.352	0.033[†]	-0.015	0.928	0.008	0.960
TNFRSF4	-0.269	0.108	0.230	0.172	0.180	0.320
UFSP1	0.018	0.916	-0.002	0.990	0.330[‡]	0.056

*Note: [†] denotes significant p-value (<0.05), [‡] denotes gene which had a PCC>0.3, but did not achieve a significant p-value. (RNA-Seq=RNA sequencing, PCC=Pearson correlation coefficient)

Supplemental Table S6: Correlation Between Gene Expression and IA CR_{stalk}.*

	Intraluminal		Proximal Parent Vessel	
**Gene**	**PCC**	**p-value**	**PCC**	**p-value**
ATF3	-0.080	0.639	-0.042	0.803
CBWD6	-0.111	0.512	-0.101	0.553
CCDC85B	0.414	0.011†	-0.253	0.132
CCR8	0.231	0.169	-0.159	0.347
CHMP4B	-0.109	0.521	-0.210	0.213
CLEC4F	0.222	0.186	-0.277	0.097
CXCL10	-0.124	0.465	-0.214	0.204
FN1	-0.067	0.693	-0.277	0.097
MT2A	-0.253	0.131	-0.099	0.560
MZT2B	-0.055	0.746	0.016	0.925
PCSK1N	0.189	0.262	-0.271	0.104
PIM3	0.206	0.221	-0.162	0.338
SLC37A3	-0.128	0.449	-0.071	0.677
ST6GALNAC1	0.294	0.077	0.156	0.356
TCN2	0.239	0.155	-0.258	0.124
TIFAB	0.319‡	0.054	-0.212	0.207
TNFRSF4	-0.012	0.943	0.182	0.281
UFSP1	0.149	0.379	-0.233	0.164

*Note: † denotes significant p-value (<0.05), ‡ denotes gene which had a PCC>0.3, but did not achieve a significant p-value. (PCC=Pearson correlation coefficient)
